# Calcified Aneurysm of the Splenic Vein

**DOI:** 10.5334/jbsr.3003

**Published:** 2022-12-12

**Authors:** Olivier Leroij, Philippe Bernard, Filip Vanhoenacker

**Affiliations:** 1 AZ Sint-Maarten and University (Hospital) Antwerp, BE; 2 AZ Sint-Maarten Mechelen, BE; 3 AZ Sint-Maarten and University (Hospital) Antwerp/Ghent, BE

**Keywords:** aneurysm, splenic vein, radiography, CT, MRI

## Abstract

**Teaching Point:** A thrombosed calcified aneurysm of the splenic vein is a rare complication of pancreatitis.

## Case History

**Figure 1:**
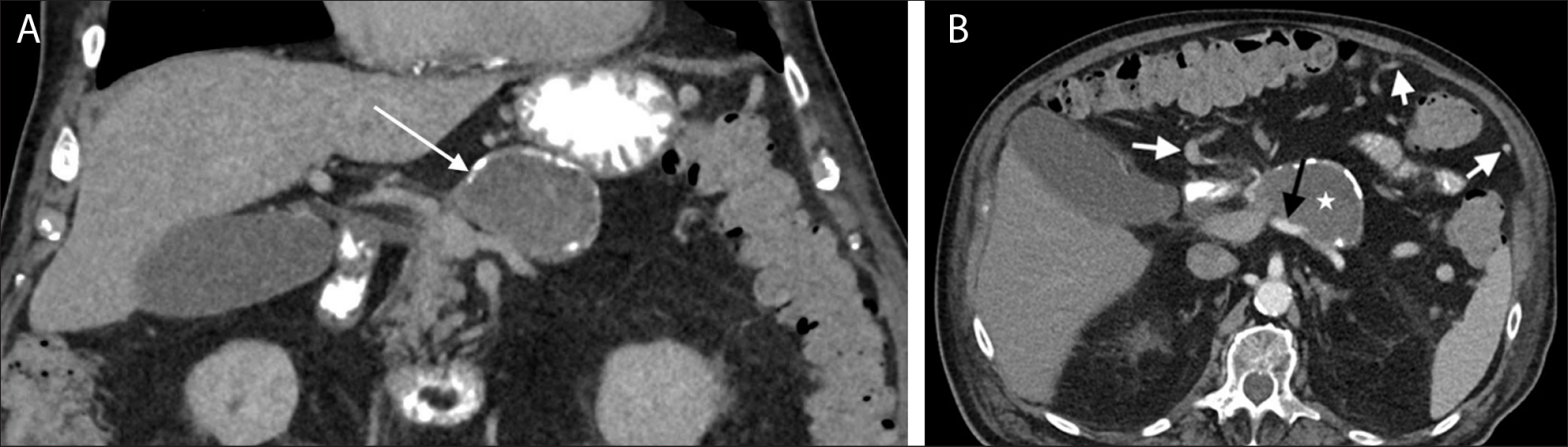


**Figure 2:**
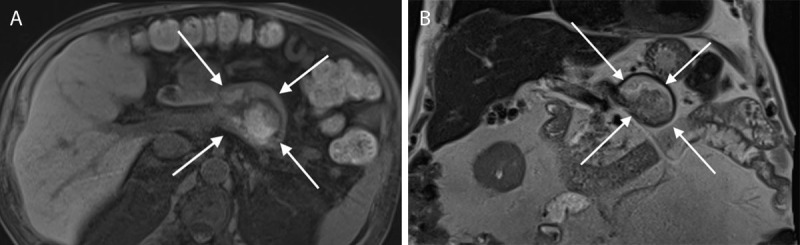


**Figure 3:**
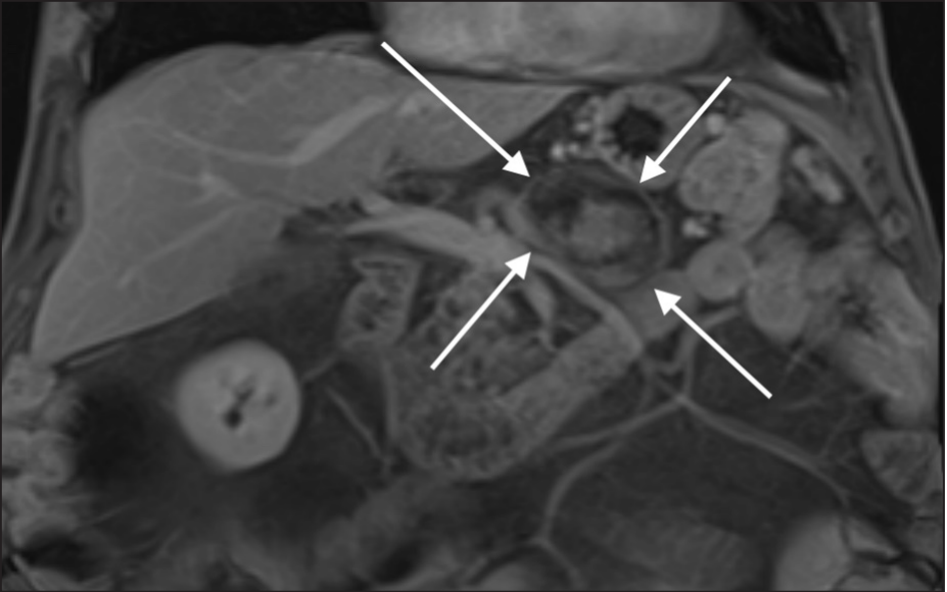


An 89-year-old man with a history of pancreatitis was admitted to the emergency department with increased inflammatory blood markers and abdominal distention. Radiography revealed an epigastric eggshell calcification. Contrast-enhanced computed tomography (CT) in the portal venous phase revealed a hypodense non-enhancing lesion with a partially calcified wall located at the level of the portal venous confluence (Figure 1A: white arrow). The splenic vein was occluded. The arterial phase showed a patent splenic artery (Figure 1B, black arrow) adjacent to the lesion (white star). Collateral circulation of the left gastroepiploic vein to the right gastroepiploic vein as well as omental branches originating from the splenic hilum was present (Figure 1B, white arrow). There was marked fatty infiltration of the pancreas. Magnetic resonance imaging (MRI) confirmed a lesion with heterogenous signal intensity on axial fat-suppressed T1-weighted images (WI) (Figure 2A, white arrows) and coronal T2-WI (Figure 2B, white arrows). The intralesional high signal on T1-WI (Figure 2A) suggests intralesional thrombus formation. There was no contrast enhancement (Figure 3). Based on these imaging findings, the diagnosis of a thrombosed splenic vein aneurysm was made.

## Comment

The prevalence of a splenic vein aneurysm is 0.43%, whereas the prevalence of a splenic artery aneurysm is up to 7–20% in patients with liver cirrhosis and portal hypertension [1].

The mechanisms by which the aneurysms develop are unknown. Inflammatory changes due to pancreatitis is the most likely cause of aneurysm formation in our patient. The origin of peripheral calcifications in venous aneurysms is still unclear.

Depending on the size of the lesion, it can remain asymptomatic or give rise to upper gastrointestinal bleeding or chronic symptoms including weight loss and/or chronic abdominal pain.

Ultrasound shows a focal saccular or fusiform, hypo- or anechoic dilatation of the splenic vein. Peripheral calcifications though interfere with visualization of the content. Turbulent flow may be observed on color Doppler.

CT confirms dilatation of the splenic vein with often delayed contrast enhancement in the portal venous phase. CT is the best technique to demonstrate peripheral calcifications.

The signal intensity on magnetic resonance imaging (MRI) and contrast enhancement depends on the absence or presence of thrombosis.

Thrombosis of a portal venous system aneurysm occurs frequently. A splenic vein thrombosis can have high attenuation on unenhanced CT, hyperintense on T1- and T2-WI and causes a filling defect after contrast administration.

Conservative treatment of thrombosed extrahepatic portal vein aneurysms provides good results [1].

## References

[1] Uy PPD, Francisco DM, Trivedi A, O’Loughlin M, Wu GY. Vascular diseases of the spleen: A review. J Clin Transl Hepatol. 2017; 5(2): 152–164. DOI: 10.14218/JCTH.2016.00062 28660153 PMC5472936

